# Supplementary material for: 1,4-Disubstituted 1H-1,2,3-Triazoles for Renal Diseases: Studies of Viability, Anti-Inflammatory, and Antioxidant Activities
Source: Int J Mol Sci. 2020 May 28;21(11):3823. doi: 10.3390/ijms21113823 (PMC7312092; doi:10.3390/ijms21113823)
Supplement: Supplementary file 1 [file ijms-21-03823-s001.pdf]

Supplementary Materials:

1,4-Disubstituted 1*H*-1,2,3-Triazoles for Renal Diseases: Studies of Viability, Anti-Inflammatory, and Antioxidant Activities

Ching-Yi Cheng [1,2,*], Ashanul Haque [3,4,*], Ming-Fa Hsieh [5,*], Syed Imran Hassan [4], Md. Serajul Haque Faizi [6], Necmi Dege [7] and Muhammad S. Khan [4]

[1] Graduate Institute of Health Industry Technology, Research Center for Chinese Herbal Medicine and Research Center for Food and Cosmetic Safety, Chang Gung University of Science and Technology, No. 261, Wenhua 1st Rd., Guishan Dist., Taoyuan City 333, Taiwan

[2] Department of Pulmonary Infection and Immunology, Chang Gung Memorial Hospital at Linkou, No. 5, Fuxing St., Guishan Dist., Taoyuan City 333, Taiwan

[3] Department of Chemistry, College of Science, University of Hail, Ha'il 81451, Kingdom of Saudi Arabia

[4] Department of Chemistry, College of Science, Sultan Qaboos University, P.O. Box 36, Al-Khod 123, Sultanate of Oman imranhassan2@gmail.com (S.I.H.); msk@squ.edu.om (M.S.K.)

[5] Department of Biomedical Engineering, Chung Yuan Christian University, No. 200 Zhongbei Rd., Zhongli Dist., Taoyuan City 320, Taiwan

[6] Department of Chemistry, Langat Singh College, B.R.A. Bihar University, Muzaffarpur, Bihar 842001, India; faizichemiitg@gmail.com

[7] Department of Physics, Faculty of Arts and Sciences, Ondokuz Mayis University, 55139 Atakum, Samsun, Turkey; necmid@omu.edu.tr

* Correspondence: jennycheng@mail.cgust.edu.tw (C.-Y.C.); a.haque@uoh.edu.sa (A.H.); mfhsieh@cycu.edu.tw (M.-F.H.)

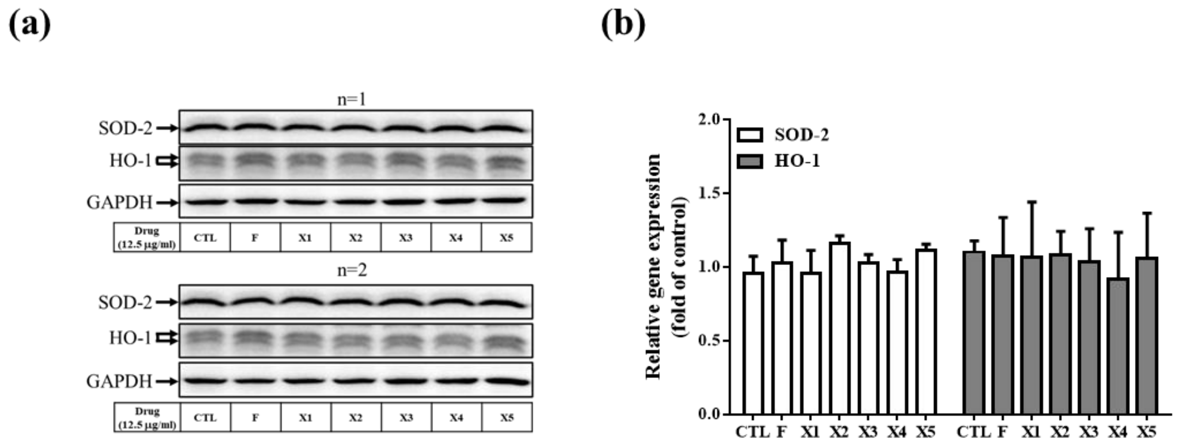

Figure S1: Effect of ferrocene-1H-1,2,3-triazole hybrids on the protein expression of SOD-2 and HO-1 in RMCs. RMCs were treated without or with F, X1, X2, X3, X4, or X5 at 12.5 μg/mL for 24 h. (**a**) The cell lysates were subjected to Western blot assay using an anti-SOD-2, anti-HO-1 or anti-GAPDH (as a control) polyclonal antibody. (**b**) The SOD-2 and HO-1 mRNA levels were determined by real-time PCR. Results are presented as the mean ± SEM of three repeated and independent assays and analyzed with one-way analysis of variance (ANOVA) and Bonferroni's multiple-comparisons test.

Table S1: Selected geometric parameters (Å, º) of compound **1.**

	**Geometric parameters from crystal structure**
Fe1—C5	2.018 (3)
Fe1—C1	2.022 (3)
Fe1—C4	2.032 (3)
Fe1—C7	2.039 (3)
Fe1—C2	2.041 (3)
Fe1—C8	2.041 (3)
Fe1—C6	2.042 (2)
Fe1—C9	2.042 (2)
Fe1—C10	2.055 (2)
N3—N2	1.354 (3)
N3—C13	1.426 (3)
N2—N1	1.307 (3)
N1—C11	1.368 (3)
O1—N4	1.212 (3)
N4—O2	1.217 (3)
N4—C16	1.471 (3)
C11—C10	1.460 (3)
C12—C11	1.358 (3)
C12—N3—N2	110.54 (18)
C12—N3—C13	128.8 (2)
N2—N3—Cl3	120.66 (18)
N1—N2—N3	106.89 (18)
N2—N1—C11	109.20 (19)
O1—N4—O2	123.6 (2)
O1—N4—C16	118.4 (2)
O2—N4—C16	118.0 (2)
O1—N4—C16—C17	1.1 (4)
O2—N4—C16—C15	1.7 (4)
C18—C13—N3—C12	17.6 (4)
C14—C13—N3—N2	16.5 (3)
C12—C11—C10—C6	-11.1 (4)
N1—C11—C10—C9	-10.5 (4)

Table S2: Selected geometric parameters (Å, º) of compound **5.**

	**Geometric parameters from crystal structure**
Fe1—C6	2.030 (2)
Fe1—C1	2.034 (2)
Fe1—C3	2.037 (2)
Fe1—C7	2.043 (2)
Fe1—C2	2.034 (2)
Fe1—C8	2.052 (2)
Fe1—C5	2.044 (2)
Fe1—C9	2.0484 (19)
Fe1—C10	2.0340 (18)
Fe1—C4	2.043 (2)
N3—N2	1.351 (2)
N3—C13	1.414 (2)
N2—N1	1.304 (2)
N1—C11	1.364 (2)
O1—N4	1.205 (2)
N4—O2	1.216 (2)
N4—C18	1.466 (2)
N3—C12	1.351 (2)
C10—C11	1.457 (3)
C12—N3—N2	109.98 (15)
C12—N3—C13	129.56 (15)
N2—N3—Cl3	120.36 (14)
N1—N2—N3	107.44 (14)
N2—N1—C11	109.20 (19)
O1—N4—O2	124.23 (18)
O1—N4—C18	117.83 (16)
O2—N4—C18	117.87 (17)
O1—N4—C18—C13	61.4 (2)
O2—N4—C18—C17	60.5 (2)
C12—N3—C13—C14	24.7 (3)
C18—C13—N3—N2	25.1 (3)
C12—C11—C10—C6	-32.6 (3)
N1—C11—C10—C9	-35.9 (3)
